# Architecture and Sanitation

**Published:** 1899-03-25

**Authors:** 


### ARCHITECTURE AND SANITATION.

In the Lumleian Lectures now being delivered before the Royal College of Physicians, Dr. Gee, speaking of the causes of bronchitis, draws attention to the infective nature of what is generally spoken of as a "common cold." We can hardly doubt, he says, that chill by exposure of the body has often something to do with it, but how? Colds are seldom caught, as we say, in pure air—at sea, for instance—no matter how great the exposure. It is also to be noted that colds are often contagious with an incubation period of about three days. This has been shown in the interesting experience of the St. Kilda cold, which also seems to show that people who are not themselves suffering from catarrh may yet convey the disease to others, and that a cold past and gone confers a temporary immunity to further attack. It has lately been stated that upon some of the small islands in Torres Strait the natives were exempt from coughs and colds until they took to wearing clothes. But it is difficult to understand how the mere fact of clothing pure and simple can have been the cause of these disorders, and it would seem more probable that the inhabitants were infected, and that the catarrhs were really due to the intruding foreigner, who brought along with him his civilisation and also his diseases.

The doctrine that most catarrhs are due to a specific infection, spreading from man to man, has very important bearings upon medical practice. It leads us to believe that the means by which we may prevent catarrh are to be found in ventilation and cleanliness, if, indeed, ventilation be not a kind of cleanliness. Experience confirms this belief. When epidemic catarrh prevails, where do we find most of our patients? In those houses which are obviously the worst ventilated, even though they be the spacious mansions of the rich. And where do our patients catch their catarrh? Either in houses of the kind just mentioned or in buildings where men most do congregate, especially in offices, shops, and churches. Large shops and stores, public museums and libraries, are ventilated as little as possible, for fear of their contents being spoiled by smoke and dust. Many churches, both in town and country, are never properly aired for another reason, namely, because their architecture does not admit of it. Those "rich windows which exclude the light" do worse than this, they exclude the air. The revival of Gothic architecture, says Dr. Gee, has been, from the sanitary point of view, a great mistake. Our despised forefathers of the eighteenth century erected plain and simple buildings, which could at least be well aired, well lighted, and kept warm and comfortable; nay, even the much ridiculed churchwarden, with his brush and pail of whitewash, was a praiseworthy minister of health. Modern dwellings are no better than the churches. In the matter of domestic sanitation people have fixed their attention too exclusively upon the drainage and the water supply; light and air are not reckoned. Many of the large red-brick houses which have been built in great numbers at the West-end of London and elsewhere during the last 25 years cannot be properly ventilated. The well of the staircase ought in every house to be a reservoir of fresh air, and to have an independent supply from without. But in many houses the staircase cannot be ventilated, except through the rooms, and, in fact, it never is ventilated. Nor are the rooms themselves much better off; their heavy mullioned windows are designed with small regard to the transmission of light and air. The subsidiary and merely ornamental arts, which do no more than please the eye, are studied to the neglect of that far greater art which promotes the happiness and welfare of the whole man—the art of preserving health.

